# Time-Dependent Comparison of the Structural Variations of Natural Products and Synthetic Compounds

**DOI:** 10.3390/ijms252111475

**Published:** 2024-10-25

**Authors:** Yi Liu, Mingzhu Cai, Yuxin Zhao, Zilong Hu, Ping Wu, De-Xin Kong

**Affiliations:** 1 State Key Laboratory of Agricultural Microbiology, Agricultural Bioinformatics Key Laboratory of Hubei Province, College of Informatics, Huazhong Agricultural University, Wuhan 430070, China; dengxue@webmail.hzau.edu.cn (Y.L.);; 2 College of Chemistry, Huazhong Agricultural University, Wuhan 430070, China

**Keywords:** physicochemical property, molecular fragment, biological relevance, chemical space, time-dependent chemoinformatic analysis

## Abstract

The identification of natural products (NPs) has played a pivotal role in drug discovery and shaped the evolution of synthetic compounds (SCs). However, the extent to which NPs have historically influenced the structural characteristics of SCs remains unclear. In this study, we conducted a comprehensive, time-dependent chemoinformatic analysis to investigate the impact of NPs on the structural evolution of SCs. The physicochemical properties, molecular fragments, biological relevance, and chemical space of the molecules from the Dictionary of Natural Products were compared in a time series fashion with a synthetic compound collection sourced from 12 databases. Our findings reveal that NPs have become larger, more complex, and more hydrophobic over time, exhibiting increased structural diversity and uniqueness. Conversely, SCs exhibit a continuous shift in physicochemical properties, yet these changes are constrained within a defined range governed by drug-like constraints. SCs possess a broader range of synthetic pathways and structural diversity, albeit with a decline in biological relevance. The chemical space of NPs has become less concentrated compared to that of SCs. In conclusion, our study suggests that the structural evolution of SCs is influenced by NPs to some extent; however, SCs have not fully evolved in the direction of NPs.

## 1. Introduction

Natural products (NPs) are essential reservoirs of innovative drug discovery [1,2]. It is widely believed that the structures of NPs are highly novel, complex, and diverse, which conveys an advantage in offering promising structures for new drug leads [3,4,5,6,7,8].

In the 1980s, the emergence of high-throughput screening (HTS) and combinatorial chemistry enabled a high yield of hits. NPs could not satisfy the demand for large quantities of compounds for HTS. Therefore, many pharmaceutical enterprises shifted their attention from NPs to synthetic compounds (SCs). However, it turned out that the advancement of combinatorial chemistry and HTS did not significantly improve the discovery rate of new molecular entities [9]. The limited structural diversity of SCs may account for the fact that HTS experiments did not produce the desired results. Hence, a renaissance of NP research occurred in the drug discovery arena [2].

As a result of a long period of natural selection [10], NPs have evolved to interact with various biological macromolecules, which implies new modes of action. According to the latest publication by Newman and Cragg on the origins of new drugs, 68% of approved small-molecule drugs between 1981 and 2019 were directly or indirectly derived from NPs [11].

NPs have historically served as a wellspring for innovative drugs, guiding the synthesis of numerous medications. Examples range from aspirin to classic nonsteroidal anti-inflammatory drugs, from mevastatin to the second-generation statins, and from penicillin to cephalosporin antibiotics, among others. Therefore, the synthesis of new chemicals is significantly influenced by NPs.

According to our analysis on some generalistic and large NP databases, the estimated total quantity of NPs stands at around 1.1 million, while the number of SCs has reached hundreds of millions. The vast dataset encourages numerous comparative analyses of NPs and SCs. Zhou et al. [12] studied NPs, SCs, and drugs in terms of their physicochemical properties and chemical space. They concluded that NPs occupy a more diverse chemical space than SCs and drugs. Medina-Franco et al. [13] reported a NP fragment library based on the COCONUT database. They compared the NP fragments with the fragments from ChEMBL and Enamine REAL, and concluded that the fragments and entire structures of NPs were more complex and diverse than the others. Ertl and Schuhmann investigated functional groups [14] and scaffolds [15] from NPs and SCs. In terms of functional groups, NPs have more oxygen atoms, ethylene-derived groups, and unsaturated systems, while SCs have more nitrogen atoms. Also, most SCs’ functional groups are chemically easily accessible. As for scaffolds found in NPs, they contain more aliphatic rings and fewer heteroatoms, except for oxygen atoms. SCs’ scaffolds contain more heteroatoms and phenyl rings. As a continuing and complementary work, Ertl’s comparative study showed that substituents of NPs and SCs present different structural features [16]. Substituents found in NPs have more oxygen atoms, stereocenters, and very few other heteroatoms, and have higher structural complexity. The substituents of SCs are rich in nitrogen and sulfur atoms, halogens, and aromatic rings. Chen et al. [17] conducted a comprehensive analysis of the ring systems of NPs and SCs. They found that NP ring systems are larger, more diverse, and more complex than those of SCs. Our group analyzed the structural features and scaffold diversity of 11 purchasable screening libraries and the Traditional Chinese Medicine Compound Database (TCMCD) [18]. We found that Chembridge, ChemicalBlock, Mucle, TCMCD, and VitasM are more structurally diverse than others, and TCMCD has the highest structural complexity. We also studied the differences between marine natural products (MNPs) and terrestrial natural products (TNPs) [19]. The results showed that, compared to TNPs, MNPs have lower solubility, more nitrogen and halogen atoms, fewer oxygen atoms, and higher molecular weight. Numerous chemoinformatic analyses have exhaustively depicted the landscape of NPs and SCs.

However, an essential issue is often ignored. That is, how has the discovery of NPs impacted the properties and structures of SCs over time? The extent to which NPs have historically influenced the structural characteristics of SCs remains unclear. Waldmann and Brakmann et al. [20,21] proposed a strategy for the design of pseudo-NPs, which involves combining two or more NP fragments by multiple arrangements not found in nature. The resulting novel structures are referred to as pseudo-NPs. These pseudo-NPs inherit the key biological relevance of NPs and occupy unprecedented chemical space or biological space, representing a human-driven branch of chemical evolution of NP structures. However, this evolution of NP structures is conceptual and does not address the fundamental issue, namely, how have the structural characteristics of NPs and SCs evolved over time?

With the aim of resolving the above puzzles, a comprehensive chemoinformatic analysis was conducted on NPs and SCs in terms of the variation of physicochemical properties, molecular fragments, biological relevance, and chemical space over time. In this study, NPs and SCs were sorted in chronological order, and then divided into groups of 5000 molecules, respectively. A total of 39 important physicochemical properties of NPs and SCs were computed and analyzed. Bemis Murcko scaffolds, ring assemblies, side chains, and retrosynthetic combinatorial analysis procedure (RECAP) fragments of NPs and SCs were generated and compared utilizing multiple metrics. In addition, the biological relevance of NPs and SCs was also evaluated. The chemical space of NPs and SCs was characterized using principal component analysis (PCA), Tree MAP (TMAP), and SAR Map to capture any potential changes. The findings of our analyses will clarify the structural variations of NPs and SCs over time and the impact of NP discovery on SC structure evolution, and provide theoretical guidance for NP-inspired drug discovery.

## 2. Results and Discussion

In total, 186,210 NPs and 186,210 SCs were included in the time-dependent comparative analysis. The molecules were sorted in early-to-late order according to their CAS Registry Numbers and grouped into 37 groups, respectively. Each group had 5000 molecules, and the remaining 1210 molecules were not included in the subsequent analysis. The corresponding annual distribution ranges of each group of molecules are shown in Table S1.

### 2.1. Physicochemical Properties

#### 2.1.1. Molecular Size

**Figure 1:**
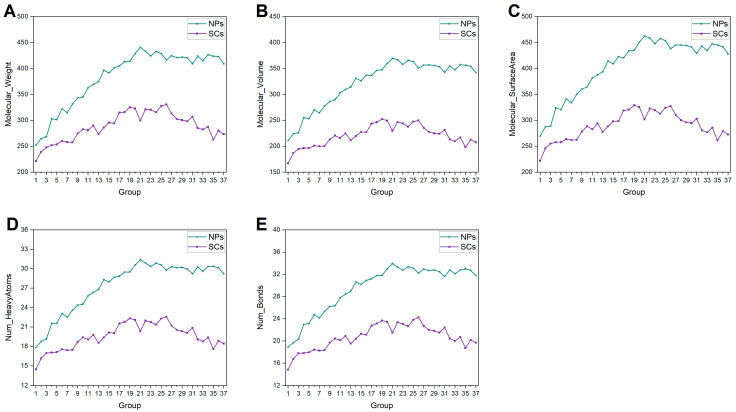
Historical changes of the molecular size-relevant properties of NPs and SCs in each group. (**A**) Molecular weight. (**B**) Molecular volume. (**C**) Molecular surface area. (**D**) Number of heavy atoms. (**E**) Number of bonds.

Five molecular descriptors were calculated to characterize the molecular size of NPs and SCs, including molecular weight, molecular volume, molecular surface area, number of heavy atoms, and number of bonds. As depicted in Figure 1, the mean values of these NPs’ descriptors exhibit a consistent increase, suggesting that recently discovered NPs tend to be larger than their early counterparts. This phenomenon can be attributed to technological advancements in separation, extraction, and purification, which enable scientists to identify larger compounds more easily. The average values of these properties of SCs vary within a limited range, which may be constrained by synthesis technology and influenced by Lipinski’s Rule of Five [22,23], especially for Group 21 (1995–1996). The data shown in Figure 1 also clearly indicate that NPs are generally larger than SCs, which is consistent with findings from earlier studies [12,17,24,25]. As the sizes of the NPs increase, this trend becomes more pronounced.

#### 2.1.2. Rings

**Figure 2:**
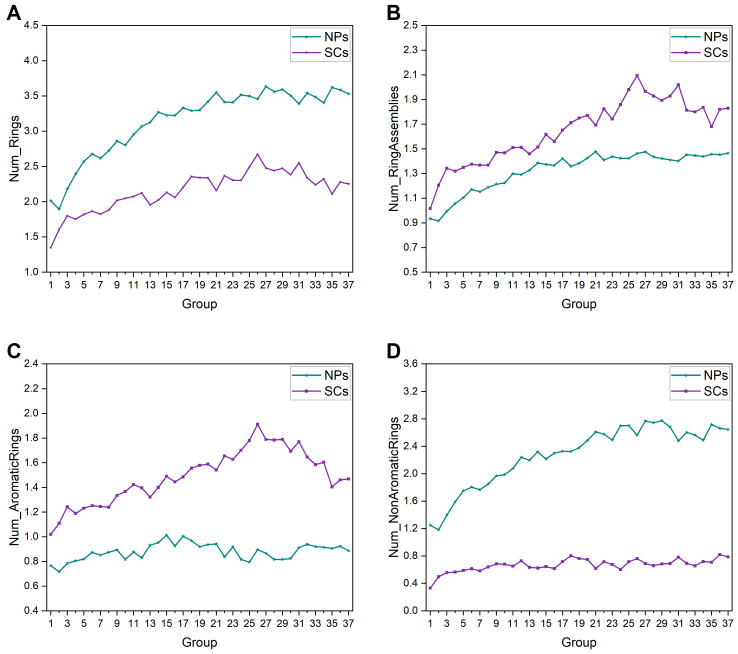
Historical changes of ring-relevant properties of NPs and SCs in each group. (**A**) Number of rings. (**B**) Number of ring assemblies. (**C**) Number of aromatic rings. (**D**) Number of nonaromatic rings.

**Figure 3:**
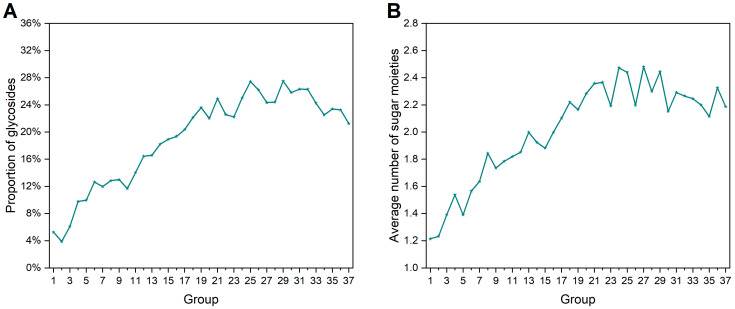
(**A**) Glycosylation ratios of each group of NPs; (**B**) the average number of sugar moieties in a glycoside per group of NPs.

The ring system is the cornerstone of the core structure and provides ample structural templates for molecular design [26]. We performed statistical analysis on properties associated with rings. In terms of NPs, the average numbers of rings, ring assemblies, and non-aromatic rings kept gradually increasing, while the average number of aromatic rings changed a little (Figure 2). In addition, as shown in Figure 3, the glycosylation ratios of each group of NPs and mean values of sugar rings in each glycoside also increased gradually over time. These results suggest that NPs are getting larger, and possess bigger fused rings (such as bridged rings and spiral rings) and more sugar rings. An evident rise is also noticeable in the mean number of rings, ring assemblies, and aromatic rings of SCs, excluding non-aromatic rings (Figure 2). Compared to SCs, NPs have more rings but fewer ring assemblies, indicating the bigger fused rings as stated above. SCs are distinguished by a greater involvement of aromatic rings, on account of the prevalent utilization of aromatic compounds such as benzene in their synthesis. In comparison, most of the rings in NPs are non-aromatic, which is consistent with a previous study [27].

The historical changes of the rings with different sizes are displayed in Figure S1. In terms of NPs, except for four-membered rings, the average numbers of other types of rings keep growing. This demonstrates that a greater number of large and complex NPs have been identified. As for SCs, the mean values of five-membered rings clearly increase and the mean values of six-membered rings are constantly high. This stems from the fact that five- and six-membered rings are stable in energy and are often introduced during the synthesis of compounds. The most surprising aspect in Figure S1 is that from Group 32 (2009) on, the average number of four-membered rings of SCs start to increase sharply. The four-membered rings can enhance pharmacokinetic properties of compounds, which makes them favored by drug designers [28]. Overall, NPs have more rings, especially large ring assemblies, than SCs, which is in line with the conclusion of Chen’s study [17].

#### 2.1.3. Molecular Polarity

**Figure 4:**
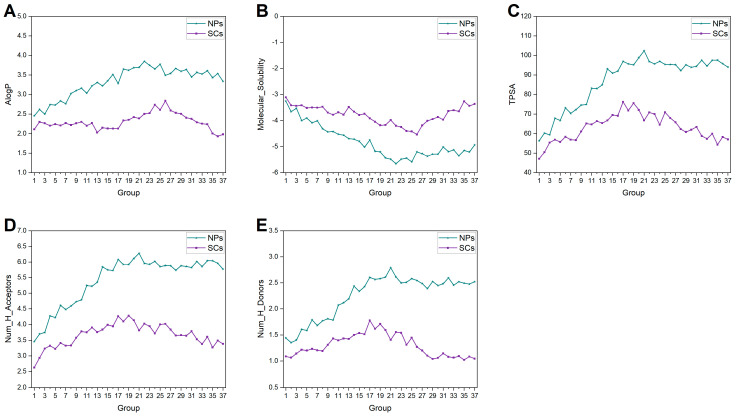
Historical changes of molecular polarity-relevant properties of NPs and SCs in each group. (**A**) AlogP. (**B**) Molecular solubility. (**C**) TPSA. (**D**) Number of hydrogen bond receptors. (**E**) Number of hydrogen bond donors.

AlogP (the Ghose and Crippen octanol–water partition coefficient) is a significant indicator in drug design [29] for contributing to the ADMET (absorption, distribution, metabolism, excretion, and toxicity) properties [30,31,32,33,34], such as solubility and permeability through membranes [30,31]. Compounds with appropriate AlogP values tend to exhibit favorable pharmaceutical characteristics [35]. As shown in Figure 4A, the mean values of NPs’ AlogP have been increasing over time. This finding can be explained by the fact that early natural product chemists predominantly relied on water-soluble extraction. Alkaloids, pigments, and organic acids were the main types of compounds found in the earliest period. Over time, there was a gradual integration of diverse organic solvents and resin separation columns into their practices. A similar phenomenon can be observed in logD (octanol–water distribution coefficient) (Figure S2).

Solubility is a critical parameter related to absorption, subsequent bioavailability, and in vitro activity of compounds [35]. NPs’ solubility exhibits a downward trend in Figure 4B. This indicates that the solubility of NPs discovered later becomes lower.

TPSA (topological polar surface area) is usually regarded as a crucial criterion for drug bioavailability [36,37,38]. In Figure 4C, the mean values of TPSA of each group of NPs keep growing, suggesting that NPs possess an increasing number of polar groups. A similar trend in terms of polar surface area can be observed in Figure S2.

Concerning hydrogen bond acceptor and hydrogen bond donor, the upward tendency can be seen in both the curves (Figure 4D,E). Taking into account that the average AlogP of the NPs is increasing at the same time, this suggests that the NPs maintain a balance of hydrophobic and hydrophilic properties, allowing for more diverse structures and greater opportunities to interact with biological macromolecules through hydrogen bonds.

However, for SCs, every molecular polarity-relevant property varies only within a limited range. This finding illustrates that no matter how much time passes, SCs are inherently designed for pharmaceutical needs, and each property should meet Lipinski’s Rule of Five or other empirical rules as closely as possible.

#### 2.1.4. Molecular Complexity

**Figure 5:**
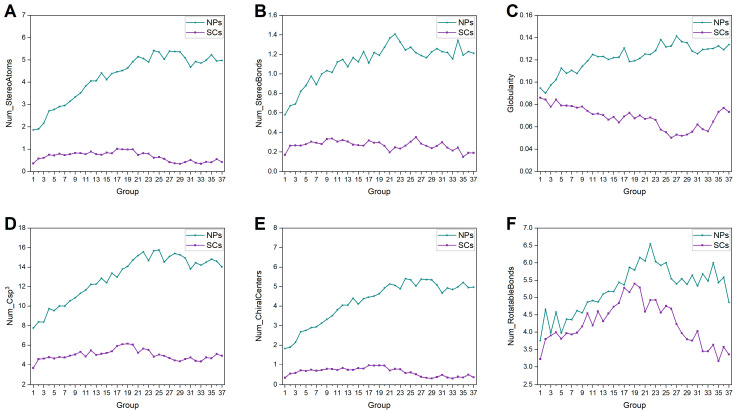
Historical changes of molecular complexity-relevant properties of NPs and SCs in each group. (**A**) Number of stereo atoms. (**B**) Number of stereo bonds. (**C**) Globularity. (**D**) Number of Csp^3^. (**E**) Number of chiral centers. (**F**) Number of rotatable bonds.

In this work, structural complexity is represented by the following properties: number of stereo atoms, number of stereo bonds, globularity (the molecular globularity ranges from 0 to 1; a value of 1 indicates a perfect sphere while a value of 0 indicates a two- or one-dimensional object), number of chiral centers, number of Csp^3^ (sp^3^ hybridized carbon atom), and number of rotatable bonds. As shown in Figure 5, the upward trend in all properties of NPs is evident, suggesting the identification of increasingly complex NPs due to technological advancements. However, the mean values of these properties for SCs remain virtually unchanged (except rotatable bonds). The rationale behind this is that excessive complexity in SCs is avoided intentionally or not, to ensure their accessibility. NPs always demonstrate higher structural complexity than SCs over time, which is in agreement with previous reports [1,24,25].

#### 2.1.5. Heteroatom Contents

**Figure 6:**
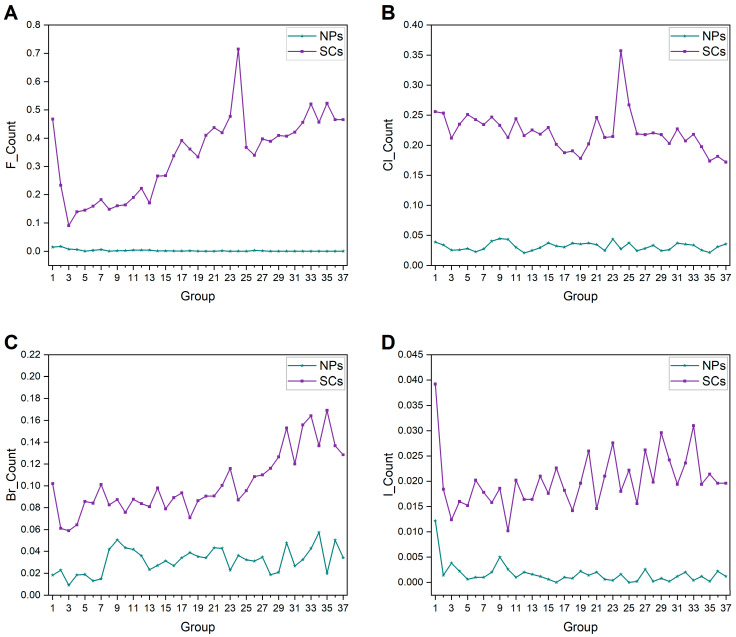
Historical changes of halogen content of NPs and SCs in each group. (**A**) F atoms. (**B**) Cl atoms. (**C**) Br atoms. (**D**) I atoms.

Typically, NPs have fewer halogens than SCs [24], which is validated in Figure 6. Over time, the average halogen content of each group of NPs shows no obvious variation. Biological systems typically have limited access to halogen atoms in their natural environments, resulting in a lower content of halogens in NPs. For SCs, the average halogen content slightly increases. What stands out in Figure 6 is that the average content of F and Cl atoms peaks in Group 24 (1999–2000) of the SCs. The distinctive properties of F and Cl atoms render them efficacious in targeting and eradicating pests; hence, they are frequently incorporated in the process of designing and synthesizing pesticides [39].

We also analyzed other elements. More detailed information can be found in Figure S3. Notably, the average content of P atoms in both NPs and SCs drops gradually. This might result from the fact that most of the NPs discovered early are water soluble and may contain phosphate groups. On the other hand, in the early stage, the heavy usage of this group in pesticides, organic reagents, and surfactants may have resulted in high levels of phosphorus atoms [40]. Afterwards, the design and use of these products were restricted due to environmental protection requirements [41].

#### 2.1.6. PCA

**Figure 7:**
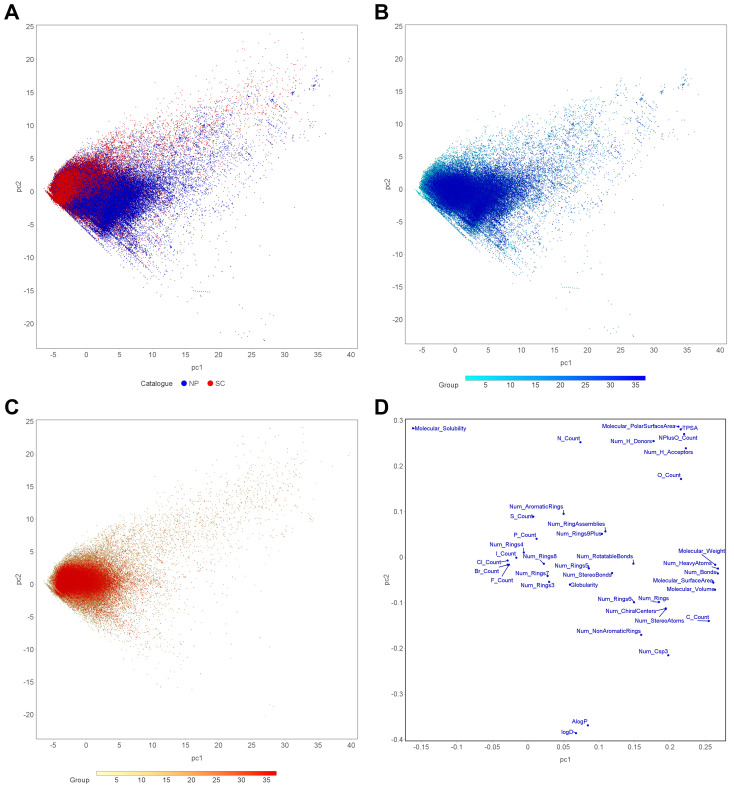
PCA based on 39 physicochemical properties for (**A**) NPs and SCs, (**B**) only NPs, and (**C**) only SCs. (**D**) The loadings plot of PC1 and PC2, which explain 34.66% and 10.89% of the total variance, respectively. The dots in panels (**B**,**C**) are colored according to the grouping information.

The chemical space occupied by the NPs and SCs was defined by the 39 physicochemical properties that describe each compound. In order to visualize these properties in two dimensions and capture historical variations of the property space, PCA was carried out. The loadings of the first two principal components are listed in Table S2. PC1 and PC2 account for 34.66% and 10.89% of the variability in the PCA data, respectively. PC1 is dominated by properties related to molecular size, such as the number of heavy atoms, number of bonds, molecular weight, molecular volume, and molecular surface area. PC2 primarily reflects molecular polarity, including molecular polar surface area, molecular solubility, and TPSA. As shown in Figure 7A, NPs populate a more extensive chemical space than SCs, which is consistent with previous research [12,17,24,25,42]. In Figure 7B, it can be observed that NPs have spread to a broader space over time. On the contrary, the chemical space of SCs becomes more concentrated over time (Figure 7C). These findings suggest that the physicochemical property space of SCs is relatively limited, which may result from Lipinski’s Rule of Five and lead-like rules in drug design. While NPs may contradict empirical rules, this contradiction is advantageous for exploring new chemical entities, as they are situated within the biologically relevant space.

### 2.2. Scaffold Analysis

To explore the variation of scaffold characteristics, we generated 167,726 NP Murcko scaffolds (32,447 are non-redundant) and 172,559 SC Murcko scaffolds (42,106 are non-redundant). About 9.34% (17,274) of NPs and 6.72% (12,441) of SCs are acyclic molecules.

Figure S4 shows that the abundance of scaffolds for NPs and SCs has been increasing and has reached around 1, indicating a growing utilization of cyclic molecules. The uniqueness of NP scaffolds has apparently been increasing; however, it remains very low (<0.16) (Figure S4). This finding suggests that there is still ample space to explore for NPs. Timely tracking and analysis of NPs is meaningful. On the other hand, the uniqueness of SC scaffolds increases significantly and is slightly higher than that of NPs. This result is due to the fact that SC libraries are designed to contain various chemotypes, which could improve hit rates in virtual screening. The scaffold novelty of NPs is characterized by slow growth and falls within the range of approximately 0.13 to 0.20 (Figure S4). This could be attributed to the fact that NPs are usually identified based on a particular class of compounds. Thus, several NPs identified may share a common scaffold. Furthermore, this is also in line with the discoveries made by Kong et al. [43], which suggest that the inherent redundancy of NPs could lead to a decrease in the proportion of new structures. The scaffold novelty of SC falls within the range of 0.17 to 0.32, which is higher than that of NPs. This phenomenon may benefit from advances in algorithmic design of compound libraries, such as de novo design and artificial intelligence.

**Figure 8:**
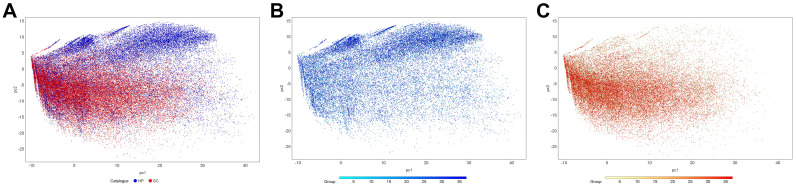
PCA using fingerprints for (**A**) NPs and SCs, (**B**) only NPs, and (**C**) only SCs. The dots in panels (**B**,**C**) are colored according to the grouping information.

Figure 8 shows the visualization of scaffold spaces generated via PCA using DataWarrior’s default fingerprint FragFp for NPs and SCs. Discrimination of chemical spaces for NPs and SCs can be easily seen in Figure 8. Compared to SCs, NPs occupy a broader chemical space, which agrees with previous perspectives [12,17,24,25]. Most newly discovered NPs are located in the upper right of the map, while recent SCs are mostly scattered in the opposite area.

### 2.3. Ring Assembly Analysis

**Figure 9:**
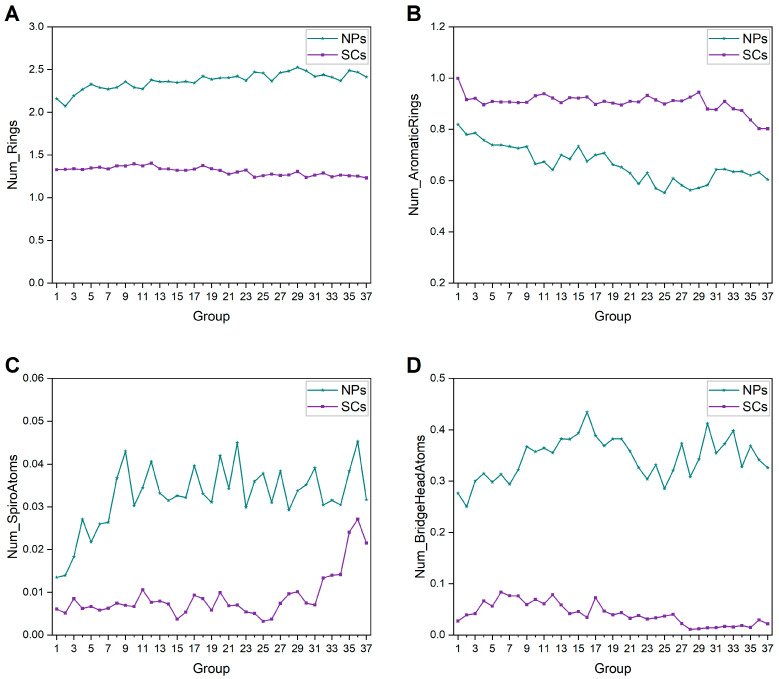
Historical changes of mean numbers of (**A**) rings, (**B**) aromatic rings, (**C**) spiro atoms, and (**D**) bridge head atoms of NP ring assemblies and SC ring assemblies in each group.

The analyzed NPs possess a total of 245,345 ring assemblies, of which 14,241 (5.80%) are non-redundant. The analyzed SCs have a total of 305,213 ring assemblies, of which 6426 (2.11%) are non-redundant. Figure 9 displays the average number of relevant ring properties (rings, aromatic rings, spiro atoms, and bridgehead atoms) for each group of ring assemblies in NPs and SCs. On average, each NP ring assembly contains two or more rings, while each SC ring assembly contains about one ring, and most of them are aromatic. Higher numbers of spiro atoms and bridge head atoms can be seen in NP ring assemblies, and they show an increasing trend. These findings suggest that NPs have more fused rings, while SCs are often dominated by individual aromatic rings.

**Figure 10:**
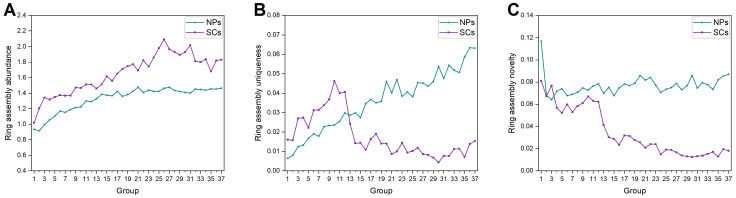
Ring assembly (**A**) abundance, (**B**) uniqueness, and (**C**) novelty of each group of NPs and SCs.

Figure 10 depicts the changes in ring assemblies of NPs and SCs. We can easily see the higher ring assembly abundance in SCs, which might result from less utilization of fused rings compared to NPs. There is a clear trend of decreasing ring assembly uniqueness and novelty in SCs. Instead, we can clearly observe the significant growth of NP ring assembly uniqueness. These findings demonstrate the capacity of NPs to provide diverse structures, and the fact that the ring assemblies introduced into SCs are limited.

**Figure 11:**
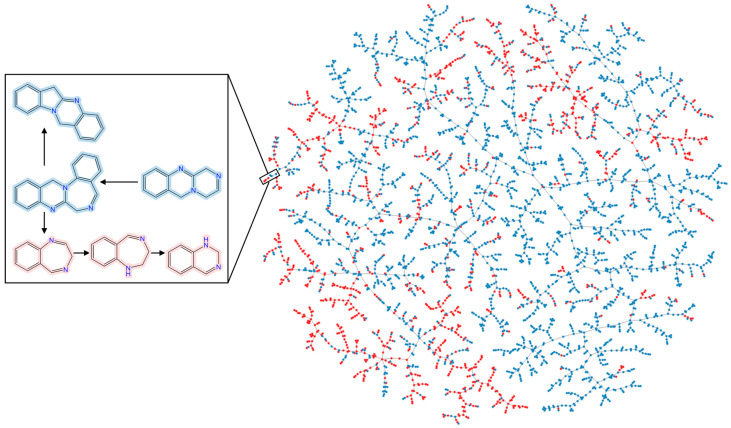
TMAP visualization of ring assemblies (frequency > 5) from NPs and SCs. NP ring assemblies are rendered blue, and SC ring assemblies are rendered red.

In order to more directly observe the impact of NPs on shaping SCs’ structures, we used TMAP to depict the chemical space of ring assemblies (frequency > 5) of NPs and SCs. TMAP can not only visualize the chemical space of molecules, but also reflect the relationships between closely related molecules in the form of a tree-like structure. In the TMAP, a point represents a ring assembly. NP ring assemblies are colored blue, and SC ring assemblies are colored red. As we can see in Figure 11, NP ring assemblies are scattered over a wider space than SCs’. Another important finding is that partial SC ring assemblies are located in the branches and sub-branches of NP ring assemblies. The chemical structures in the box of Figure 11 serve as an example. This illustrates that more complex fused rings can be found in NPs’ ring assemblies, and SCs’ structures may be influenced by NPs to some extent.

**Figure 12:**
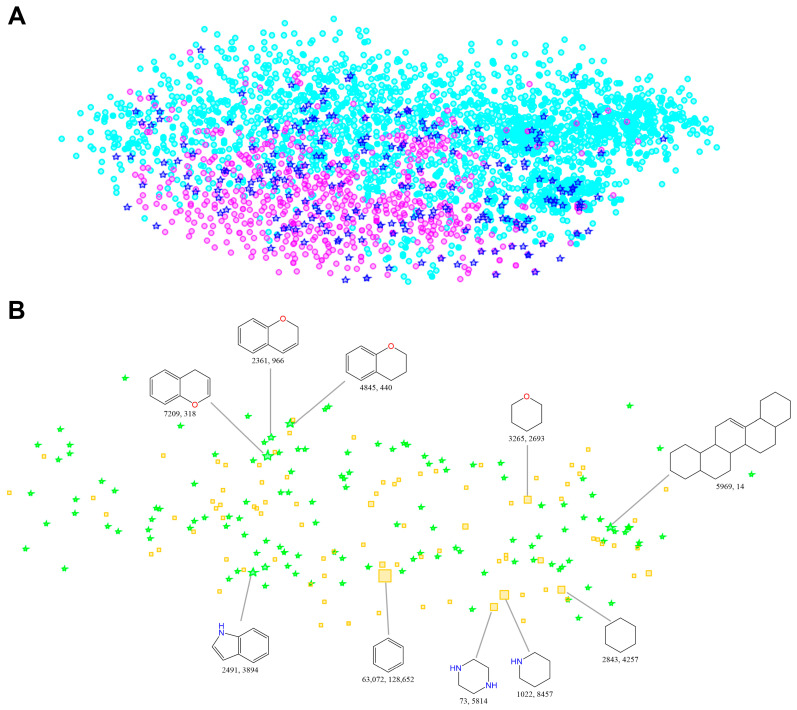
(**A**) The SAR Map of non-redundant ring assemblies (frequency > 5) extracted from NPs and SCs. Cyan circles represent NP unique ring assemblies. Magenta circles represent SC unique ring assemblies. Blue pentagrams represent common ring assemblies shared by NPs and SCs. One point represents a ring assembly. (**B**) An enlarged section of the SAR Map of common ring assemblies occurring first in NPs (green pentagrams) and SCs (yellow squares). One point represents a ring assembly. The point size reflects the frequency of a ring assembly. The top 5 most frequent common ring assemblies occurring first in NPs or SCs are shown. The first numerical value indicates the frequency of the ring assembly in NPs, and the latter represents the frequency of the ring assembly in SCs.

We also generated a SAR Map to characterize the chemical space distribution of NP ring assemblies and SC ring assemblies. The SAR Map can map structurally similar molecules into adjacent areas using nonlinear mapping. Generally, in the SAR Map, a point represents a molecule. The color and shape of the point are usually used to represent different classes of molecules. Not surprisingly, as shown in Figure 12A, NP ring assemblies dominate the chemical space of all ring assemblies. Common ring assemblies unite NP unique ring assemblies and SC unique ring assemblies. In Figure 12B, the point sizes reflect the frequencies of ring assemblies. We can see that the most frequent ring assemblies of NP-first common ring assemblies (i.e., common ring assemblies occurring first in NPs) are mostly fused rings, and often contain oxygen atoms. They occur mainly in NPs and less in SCs. By contrast, the most frequent structures of SC-first common ring assemblies (i.e., common ring assemblies occurring first in SCs) are five- or six-membered monocyclic rings, and they often have nitrogen atoms.

### 2.4. Side Chain Analysis

**Figure 13:**
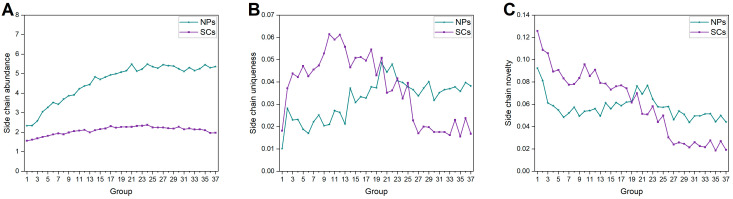
Side chain (**A**) abundance, (**B**) uniqueness, and (**C**) novelty of each group of NPs and SCs.

A total of 849,850 NP side chains were generated, of which 10,640 were non-redundant. As for SCs, there were 386,591 side chains, of which 11,246 were non-redundant. Only 1287 types of side chain are shared by NPs and SCs, suggesting different chemical modifications between them. As shown in Figure 13A, the abundance of side chains in NPs gradually increases, indicating that the structures of NPs become more spherical. This finding is consistent with Figure 5C. Furthermore, the uniqueness of NP side chains shows a clear increase (Figure 13B). Although the novelty of NP side chains has slightly decreased, it still remains within a certain range (Figure 13C). These results demonstrate that the uniqueness and novelty of NP side chains remain advantageous for producing diverse structures. Considering the side chains of SC, a relatively low level of abundance implies simple spatial structures. What is striking in Figure 13 is that the uniqueness and novelty of SC side chains drop sharply. During compound design and synthesis, the introduction of side chains is more empirically oriented and is characterized by a high utilization of chemically easily available fragments, such as halogens, hydroxy groups, and methyl groups.

### 2.5. RECAP Fragment Analysis

The RECAP analysis fragments a molecule by breaking certain bonds that are estimated to be those that can be reformed by common reliable chemistry. The resulting fragments are termed RECAP fragments. The analyzed NPs in this study provided 178,980 RECAP fragments, of which 41,044 were non-redundant. The analyzed SCs offered 236,502 RECAP fragments, with 45,540 being non-redundant. This phenomenon is consistent with the results of the above scaffold analysis. It indicates that the fragments utilized in compound synthesis are limited, resulting in a well-defined chemical space. Figure S5 shows that the abundance of SC RECAP fragments increases significantly, which is due to the flexible design of the synthesis routes. Another finding is that the uniqueness and novelty of NPs and SCs in RECAP fragments show a similar trend of continuous growth, implying diverse synthetic pathways.

### 2.6. Biological Relevance Analysis

**Figure 14:**
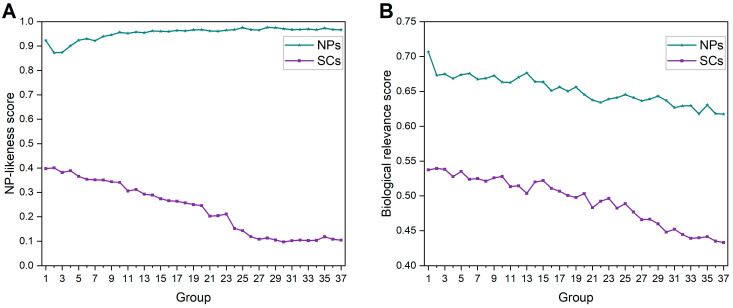
(**A**) Average NP-likeness scores for each group of NPs and SCs; (**B**) average BR scores for each group of NPs and SCs.

We calculated the average NP-likeness scores and average biological relevance scores (BR scores) to evaluate the biological relevance for each group of NPs and SCs. As shown in Figure 14A, NPs have higher NP-likeness scores, which are relatively stable within the range of 0.87 to 1. SCs have lower NP-likeness scores than NPs, and these scores gradually decrease. In addition, the BR scores of SCs are lower than those of NPs. Also, SCs show a more pronounced decrease in BR scores compared to NPs (Figure 14B). These phenomena reflect that, during compound synthesis, some compounds are often directly designed based on active pharmacophores or molecules, without incorporating many features from NPs. This trend was accelerated by the strategy change to combinatorial chemistry and HTS in the 1980s. In light of this, the influence of NP discovery on the structural variations of SC does not seem significant.

## 3. Materials and Methods

### 3.1. Data Sets and Processing

In this study, two sets of compounds were assembled for the time-dependent analysis of NPs and SCs. All the NPs were extracted from the Dictionary of Natural Products (version 2016, DNP) [44], which is the most comprehensive NP database. Only the NPs annotated with CAS Registry Numbers and explicit structures were retained. NP structures were standardized, including keeping the largest fragment, adding hydrogen atoms, organic filtering, and removing very small molecules (molecular weight < 70 Da) and large molecules (molecular weight > 2000 Da). About 16% of NPs are glycosylated [27], where sugar moieties are attached to the intrinsic structures of the NPs through glycosidic bonds. Glycosylation primarily improves the pharmacokinetic parameters of the molecule and only rarely affects bioactivity [45]. Here, we used the open-source software Sugar Removal Utility (https://github.com/JonasSchaub/SugarRemoval, accessed on 31 August 2023) for the detection and removal of sugar moieties [46]. Two parameters were adjusted: both terminal and non-terminal sugars were removed; the exocyclic oxygen atoms to atoms in ring ratio threshold was set as 0.4. Other parameters were set to their default values. After implementing the in silico deglycosylation, the deglycosylated NP structures were kept for the subsequent analysis. As a result, 187,905 NPs are annotated with CAS Registry Numbers and have explicit structures. During the process of deglycosylation, 1695 NPs were completely removed as they were basically sugars. Therefore, 186,210 NPs were kept in full for subsequent analyses.

Due to the limited availability of SC repositories with CAS Registry Numbers, we collected and curated data from various sources to compile the SC dataset. An overview of SC databases used in this study is listed in Table 1. Molecules without CAS Registry Numbers were discarded. Considering the fact that there may be labeling errors in the compound classification, we excluded the SCs that had the same structure as known NP structures. First, we selected the DNP (version 2016) and the COCONUT (January 2022) [47] as the reference for known NP structures. Then, any structures that appeared in both the reference data set and SC data set were removed from the latter. Furthermore, in order to ensure that the SCs for subsequent analysis were at the same or adjacent time as NPs, a matched number of compounds was selected from the SC data set with the smallest difference in CAS Registry Numbers compared to the NPs. Finally, the selected 186,210 SCs were standardized in the same manner as the NPs. Due to the very low glycosylation ratio of SCs [27], we did not perform the deglycosylation process on the SCs.

All of the above preprocessed NPs and SCs were sorted separately in early-to-late order according to the CAS Registry Numbers. Every 5000 molecules were separated into a group. All standardization and calculations in this study were done with Pipeline Pilot (version 2016, PP) [48].

### 3.2. Calculation of Physicochemical Properties

Physicochemical properties are typically used to describe molecular characteristics. In order to depict the historical features of NPs and SCs over time, we calculated 39 physicochemical properties related to molecular size, molecular polarity, molecular complexity, and heteroatoms (see Table S3). The calculation of Globularity, Num_ChiralCenters, Num_HeavyAtoms, and TPSA was conducted with the MOE modeling suite (version 2019.0102) [49]. Other properties were calculated in PP. The statistical results (mean values of properties for each group of compounds) were obtained using the component “Basic Statistics by Category” in PP.

### 3.3. Generation of Fragment Representations

Four types of molecular fragments were generated to portray the structural characteristics of NPs and SCs, namely Bemis Murcko scaffolds, ring assemblies, side chains, and RECAP fragments. Bemis Murcko scaffolds are defined as contiguous ring systems plus linkers between them without side chains [50], which represent the core structure of a molecule. Molecular scaffolds were generated with the “Generate Fragments” component (with the parameter set as BemisMurckoAssemblies) in PP. After obtaining the scaffolds of the molecules, we implemented “Generate RGroups” in PP to collect side chains of molecules. The scaffolds were mapped onto the original molecules, generating fragments in which the attachment points are marked as template (*Z*) atoms. The fragments with template atoms are side chains of the molecules. Ring assembly refers to the contiguous ring systems of a molecule. Ring assemblies were also generated by the component “Generate Fragments” in PP (with the parameter set as RingAssemblies). The RECAP fragments were generated by using the “Generate RECAP Fragments” module in Discovery Studio (version 2018) [51]. Only the fragments with a number of heavy atoms greater than three were kept.

### 3.4. Analysis of Fragment Representations

To comprehensively evaluate the structural characteristics of molecular fragments, we defined three metrics: abundance, uniqueness, and novelty.

The fragment abundance is defined as:

(1) $$\mathrm{fragment}\;\mathrm{abundance}=\frac{N}{M}$$

N: Total number of fragments in each group;

M: Total number of molecules in each group.

The fragment uniqueness is defined as:

(2) $$\mathrm{fragment}\;\mathrm{uniqueness}=\frac{N_{\mathrm{sing}}}{M}$$

N_sing_: Total number of singleton fragments in each group (singleton fragments only appear one time in the whole data set);

M: Total number of molecules in each group.

The fragment novelty is defined as:

(3) $$\mathrm{fragment}\;\mathrm{novelty}=\frac{N_{\mathrm{novel}}}{M}$$

N_novel_: Total number of novel fragments in each group that never occur in previous groups;

M: Total number of molecules in each group.

### 3.5. Evaluation of Biological Relevance

It is widely believed that NPs possess an inherent capability to interact with biological targets during the long period of evolution, and occupy the biologically relevant chemical space. Higher biological relevance contributes to surviving the drug development pipelines [52]. Therefore, in this study, we utilized two metrics to measure the biological relevance of the compounds, and to assess the impact of NPs on SCs. First, we completed the calculation of the NP-likeness score for NPs and SCs through the NP-Scout (https://nerdd.univie.ac.at/npscout/, accessed on 9 April 2024) [53,54]. Then, the average NP-likeness scores were calculated for each group of compounds. Next, the similarities between the object molecules and the Biorelevant Representative Compounds Database compounds (termed as BR scores) [52] were calculated. Average BR scores for each group of compounds were also calculated.

### 3.6. Chemical Space Visualization

#### 3.6.1. PCA

PCA is widely used in chemical space visualization. It is a statistical technique used for dimensionality reduction by transforming a large set of variables into a smaller set that still contains most of the original information. PCA achieves this by identifying the directions (principal components) in which the data varies the most, and projecting the data onto these components. In this work, DataWarrior (version 5.5.0) [55] was employed to conduct PCA to representing (a) the property space using the 39 physicochemical properties, and (b) the scaffold space based on DataWarrior’s default fingerprint FragFp of NPs and SCs.

#### 3.6.2. TMAP

TMAP is a way of visualizing high-dimensional data sets in the form of minimum-spanning trees [56]. It preserves local features (i.e., relationships) between the close neighbor molecules. We applied TMAP (http://tmap.gdb.tools, accessed on 24 April 2024) to visualize the chemical space of ring assemblies. Reduplicative ring assemblies of NPs and SCs were removed, respectively. The ring assemblies with frequency ≤5 were filtered. The remaining ring assemblies were utilized to generate TMAP. The point size was set as 4. Other parameters were default.

#### 3.6.3. SAR Map

The SAR Map is also a good way to visualize the chemical space for a collection of compounds. It organizes similar molecules or fragments into close locations or clusters using nonlinear mapping. In this work, the SAR Maps were generated with the DataMiner 1.6 software [57]. The ring assemblies used to generate the SAR Map were the same as the ones for the above TMAP. These ring assemblies were divided into three categories: common ring assemblies (occurring in both NPs and SCs), NP-unique ring assemblies (occurring only in NPs), and SC-unique ring assemblies (occurring only in SCs). Based on the CAS Registry Numbers of compounds, common ring assemblies were further divided into two categories: common ring assemblies occurring first in NPs (NP-first common ring assemblies) and common ring assemblies occurring first in SCs (SC-first common ring assemblies). The SAR Map based on these ring assemblies was generated by the “Generate SAR Map” function in DataMiner 1.6. All of the parameters were set to their default values.

## 4. Conclusions

The chemical space of small molecules has been estimated to be up to 10^60^ [58]. However, only a small fraction of these molecules can bind to biomolecular targets and exhibit biological activity, which tends to cluster near the NP space. Consequently, scientists have introduced various “natural product-likeness” or “biological relevance” concepts to significantly enhance the efficiency of drug screening.

Historically, NPs have been a major source of innovation in drug discovery [11]. Many drugs or drug candidates have been synthesized under the guidance of NPs, as mentioned in the introduction. Clearly, the identification of NPs has played a pivotal role in shaping the evolution of SCs. The objective of this study was to investigate the temporal structural variations of NPs and SCs, and elucidate the impact of NP discovery on the development of SCs.

The DNP database and SC data set composed of multiple databases were analyzed using a series of time-dependent cheminformatic methods. Physicochemical property analysis indicates that NPs become larger, more lipophilic (with lower water solubility), more complex, and have more non-aromatic rings (especially sugar rings) and fused rings over time. Analyses of molecular fragments indicate that NPs inherently have the ability to produce diverse structures. Biological relevance analysis shows the advantages of NPs in interacting with biological targets. As for SCs, the average values of molecular size, ring, polarity, and heteroatom-relevant properties vary within a well-defined range. Fragment analyses show that SCs are often monocyclic and aromatic (such as benzene), and have considerable structural diversity. However, the biological relevance of SCs drops significantly.

Taken together, these results show that both NPs and SCs exhibit regular structural changes, with molecules becoming larger, more complex and more diverse. Furthermore, NPs do indeed have some influence on the SC structural changes (Figure 11). Despite the various “natural product-like” concepts, the structures and properties of SCs have not fully evolved in the direction of NPs. As an example, current SCs are shifting away from NP-like or biologically relevant chemical space (Figure 14). This paradoxical phenomenon seems to suggest that the chemical space of NPs has not been fully utilized. The design and synthesis of compounds should take more inspiration from NPs, which could potentially lead to improved success rates in drug discovery. However, some practical factors (such as synthetic accessibility, reaction yields, chemical stability, costs, etc.) restrict efforts in multi-step syntheses that would be necessary to generate large libraries of NP-like compounds. The practical restraints will also be a factor when synthetic chemists are considering using NPs as inspiration.

However, in this study, we used CAS Registry Numbers to determine the discovery or synthesis year of the compounds. Despite the large number of molecules, the limitation of available NPs and SCs with CAS numbers makes the study more akin to a sample analysis. As a result, the full impact of NPs on shaping SC structures may not be fully reflected. For those interested in the discovery history of a particular class of compounds or molecular scaffolds, a more detailed analysis incorporating additional compounds with CAS numbers would likely provide more compelling evidence.

## Figures and Tables

**Table 1:** Overview of the sources of synthetic compounds.

Database	Version	Size	Number of Molecules with CAS Registry Number
ChEMBL	32	2,327,928	66,248
Alinda ^a^	2020	202,332	11,034
ChEBI	2023	151,162	21,634
Maybridge ^a^	2021	53,352	6239
NCI	2012	265,242	125,723
LI ^a^	2012	1,427,028	267,004
LN ^a^	2017	320,336	306,779
BIONET ^a^	2020	212,374	211,263
HTS Biochemie Innovationen ^a^	2021	52,460	4113
INDEX-NET ^a^	2004	78,730	61,949
Lifechemicals ^a^	2021	474,738	454,591
In-house database ^b^	2008	575,468	519,723

^a^ Provided by Topscience (https://www.tsbiochem.com/, accessed on 23 April 2023). ^b^ An in-house database consisting of multiple sources of compounds with CAS Registry Numbers. Before the analysis, all natural products were removed.

## Data Availability

The original contributions presented in the study are included in the article/Supplementary Material; further inquiries can be directed to the corresponding author.

## Supplementary Materials

The supporting information can be downloaded at: https://www.mdpi.com/article/10.3390/ijms252111475/s1.
