# Supplementary material for: Time-Dependent Comparison of the Structural Variations of Natural Products and Synthetic Compounds
Source: Int J Mol Sci. 2024 Oct 25;25(21):11475. doi: 10.3390/ijms252111475 (PMC11547171; doi:10.3390/ijms252111475)

Time-Dependent Comparison of the Structural Variations of Natural Products and Synthetic Compounds

Yi Liu[1], Mingzhu Cai[1], Yuxin Zhao[1], Zilong Hu[1], Ping Wu[2], De-Xin Kong[1*]

[1]State Key Laboratory of Agricultural Microbiology, Agricultural Bioinformatics Key Laboratory of Hubei Province, College of Informatics, Huazhong Agricultural University, Wuhan 430070, P. R. China

[2]College of Chemistry, Huazhong Agricultural University, Wuhan 430070, P. R. China

[*]Corresponding author

De-Xin Kong

College of Informatics

Huazhong Agricultural University,

Shizishan Str. 1#, Hongshan Dist.

Wuhan 430070, China

Tel.: +86-27-8728 0877

Email: dxkong@mail.hzau.edu.cn

Table S1. The annual distribution of the compounds in each group

Group No.	Year	Group No.	Year
1	1965-1965	20	1993-1995
2	1965-1965	21	1995-1996
3	1965-1966	22	1996-1998
4	1966-1968	23	1997-1999
5	1968-1969	24	1999-2000
6	1969-1972	25	2000-2001
7	1972-1974	26	2001-2003
8	1974-1976	27	2002-2004
9	1976-1978	28	2004-2005
10	1978-1980	29	2005-2006
11	1980-1981	30	2006-2007
12	1981-1983	31	2007-2008
13	1983-1985	32	2009-2009
14	1985-1986	33	2009-2010
15	1986-1988	34	2009-2011
16	1987-1989	35	2011-2012
17	1989-1991	36	2012-2012
18	1990-1992	37	2012-2014
19	1992-1993	38*	2014-2016

*Group 38 has only 1210 molecules, less than 5000. Therefore, this group was omitted.

Table S2. Loadings of the first two principal components of the property space

Variable name	PC1	PC2
AlogP	0.0845	-0.3687
Molecular_Weight	0.2638	-0.0165
Molecular_Solubility	-0.1616	0.2831
logD	0.0678	-0.3858
Molecular_Volume	0.2637	-0.0713
Molecular_SurfaceArea	0.2612	-0.0554
Molecular_PolarSurfaceArea	0.2121	0.2867
Num_Bonds	0.2675	-0.0354
Num_RotatableBonds	0.1487	-0.0139
Num_Rings	0.1846	-0.0982
Num_AromaticRings	0.0504	0.0953
Num_RingAssemblies	0.1093	0.0568
Num_Rings3	0.0299	-0.0539
Num_Rings4	-0.0055	0.0103
Num_Rings5	0.0860	-0.0241
Num_Rings6	0.1496	-0.0989
Num_Rings7	0.0279	-0.0409
Num_Rings8	0.0227	-0.0140
Num_Rings9Plus	0.1045	0.0514
Num_StereoAtoms	0.1939	-0.1136
Num_StereoBonds	0.1187	-0.0347

Num_H_Acceptors	0.2226	0.2392
Num_H_Donors	0.1772	0.2550
F_Count	-0.0265	-0.0163
Cl_Count	-0.0284	-0.0073
Br_Count	-0.0286	-0.0163
I_Count	-0.0163	-0.0010
C_Count	0.2548	-0.1396
N_Count	0.0741	0.2524
O_Count	0.2158	0.1720
S_Count	0.0078	0.0891
P_Count	0.0124	0.0407
NPlusO_Count	0.2201	0.2705
Num_NonAromaticRings	0.1596	-0.1701
Num_Csp^3	0.1977	-0.2150
Num_HeavyAtoms	0.2676	-0.0248
Num_ChiralCenters	0.1942	-0.1122
Globularity	0.0593	-0.0599
TPSA	0.2154	0.2806

Table S3. 39 physicochemical properties used to analyze the natural products (NPs) and synthetic compounds (SCs)

**Descriptor abbreviations**	**Descriptors**
AlogP	Octanol-water partition coefficient
Globularity	Globularity
logD	Distribution coefficient
Molecular_PolarSurfaceArea	Molecular polar surface area
Molecular_Solubility	Molecular solubility
Molecular_SurfaceArea	Molecular surface area
Molecular_Volume	Molecular volume
Molecular_Weight	Molecular weight
Num_H_Acceptors	Number of hydrogen bond acceptors
Num_AromaticRings	Number of aromatic rings
Num_Bonds	Number of bonds
Num_Br_Atoms	Number of bromine atoms
Num_C_Atoms	Number of carbon atoms
Num_ChiralCenters	Number of chiral centers
Num_Cl_Atoms	Number of chlorine atoms
Num_Csp^3	Number of sp^3 hybridized carbon atoms
Num_F_Atoms	Number of fluorine atoms
Num_H_Donors	Number of hydrogen bond donors
Num_HeavyAtoms	Number of heavy atoms
Num_I_Atoms	Number of iodine atoms

Num_N_Atoms	Number of nitrogen atoms
Num_NonAromaticRings	Number of nonaromatic rings
Num_NPlusO_Atoms	Number of nitrogen and oxygen atoms
Num_O_Atoms	Number of oxygen atoms
Num_P_Atoms	Number of phosphorus atoms
Num_Rings	Number of rings
Num_Rings3	Number of three-membered rings
Num_Rings4	Number of four-membered rings
Num_Rings5	Number of five-membered rings
Num_Rings6	Number of six-membered rings
Num_Rings7	Number of seven-membered rings
Num_Rings8	Number of eight-membered rings
Num_Rings9Plus	Number of over nine-membered rings
Num_RingAssemblies	Number of ring assemblies
Num_RotatableBonds	Number of rotatable bonds
Num_S_Atoms	Number of sulfur atoms
Num_StereoAtoms	Number of stereo atoms
Num_StereoBonds	Number of stereo bonds
TPSA	Topological polar surface area

Figure S1. Historical changes of the number of rings with different sizes of the NPs and SCs in each group.

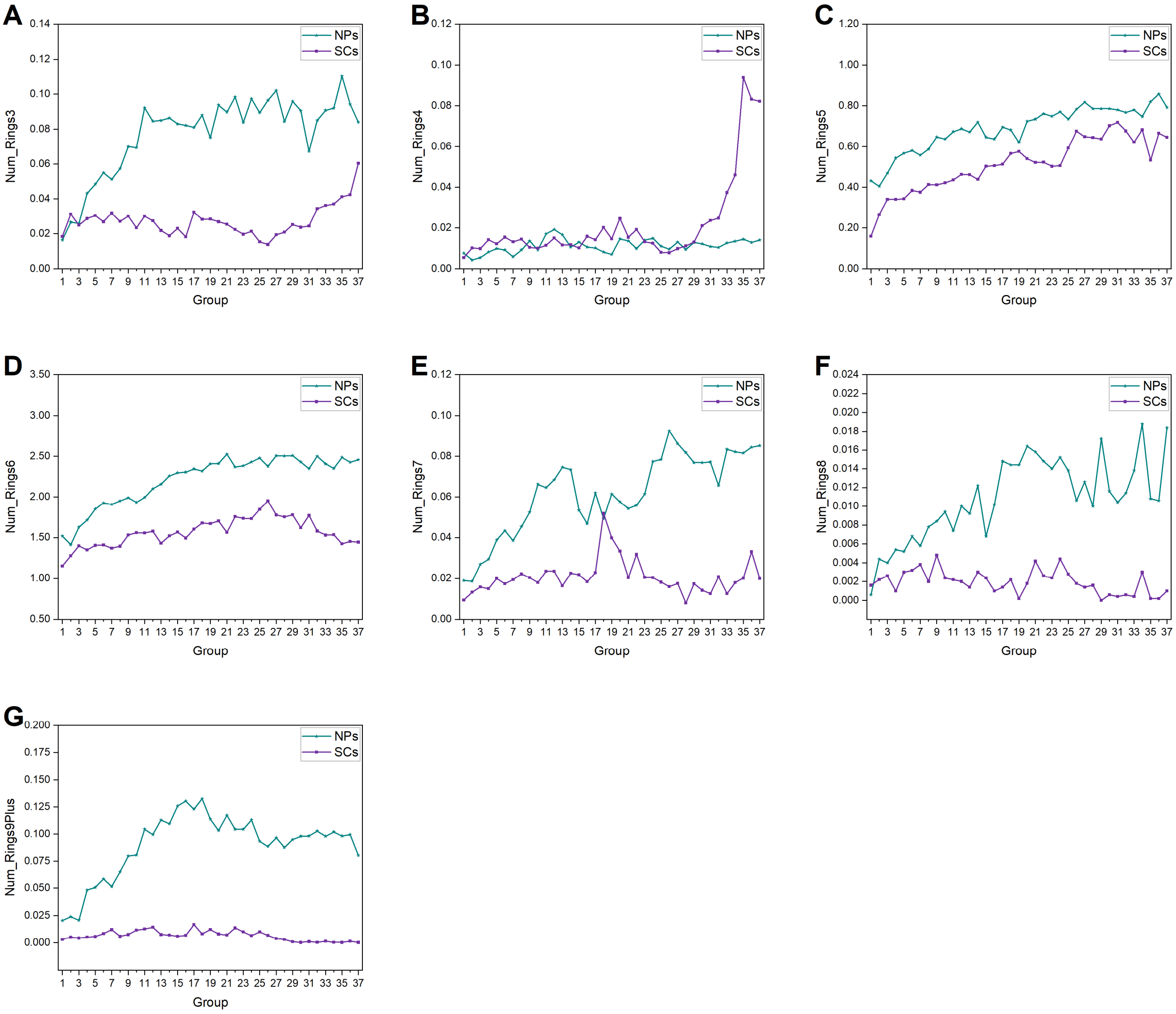

Figure S2. Historical changes of other molecular polarity-relevant properties of the NPs and SCs in each group.

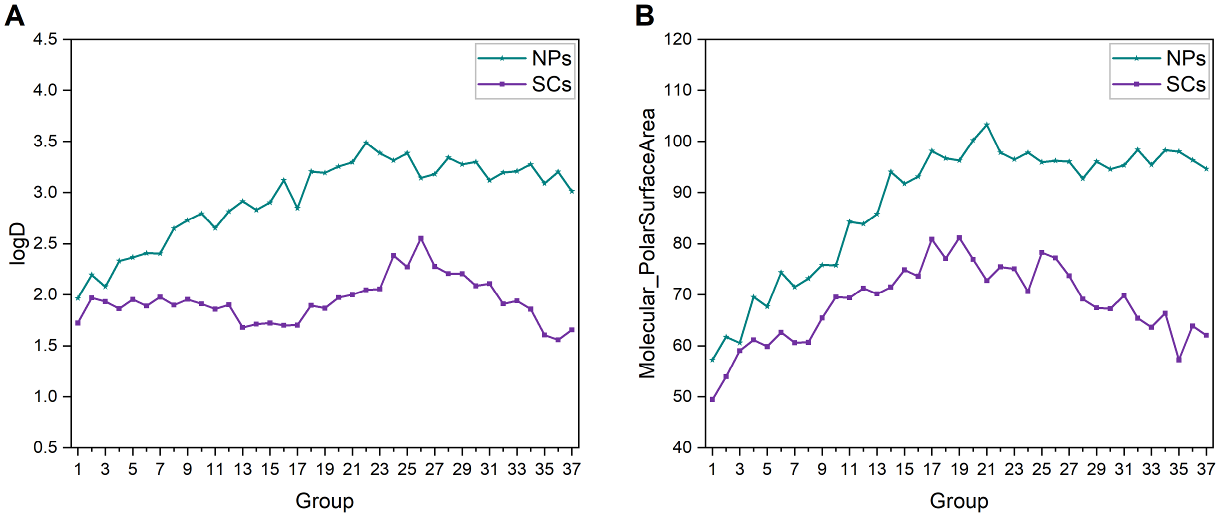

Figure S3. Historical changes of other atom contents of the NPs and SCs in each group.

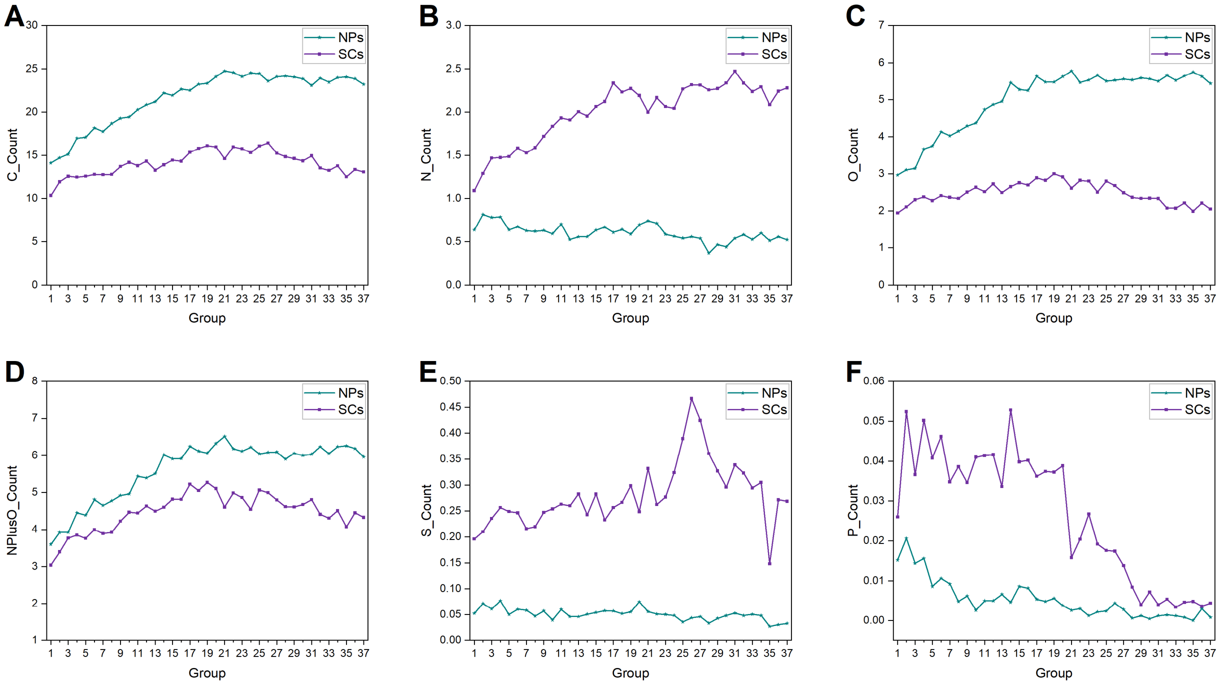

Figure S4. Scaffold **(A)** abundance, **(B)** uniqueness, and **(C)** novelty of each group of NPs and SCs.

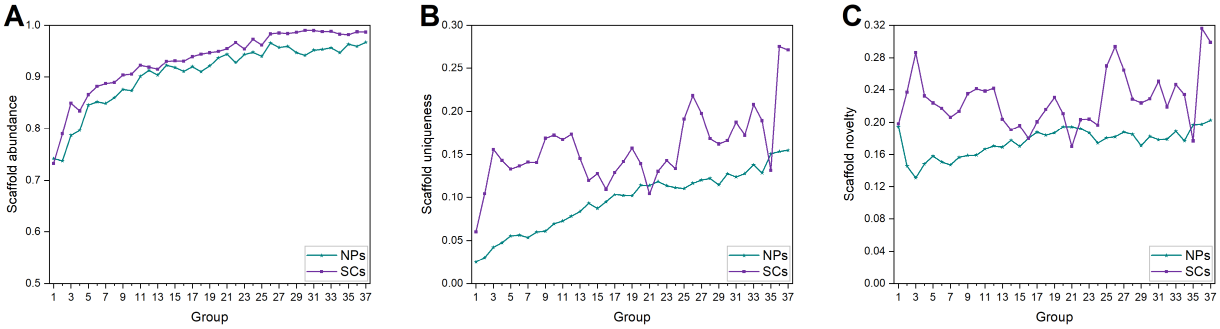

Figure S5. RECAP fragment **(A)** abundance, **(B)** uniqueness, and **(C)** novelty of each group of NPs and SCs.

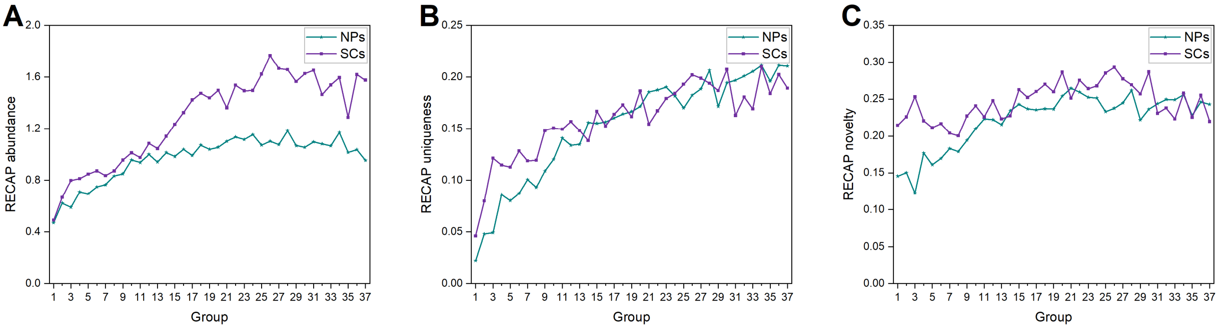

Supplement: Supplementary file 1 [file ijms-25-11475-s001.zip › ijms-3195075-supplementary.pdf]
